# Preparation, Thermal, and Optical Properties of D-A-Type Molecules Based on 1,3,5-Triazine for Violet-Blue Fluorescent Materials

**DOI:** 10.3390/ma18092043

**Published:** 2025-04-29

**Authors:** Lu Wang, Enwang Du, Zhi Liu, Zhiqiang Liu

**Affiliations:** 1 State Key Laboratory of Crystal Materials and Institute of Crystal Materials, Shandong University, Jinan 250100, China; 2 School of Physics, Shandong University, Jinan 250100, China

**Keywords:** triazine, D-A-type molecules, fluorescent materials, violet emission

## Abstract

Organic violet-blue fluorescent materials have garnered significant interest for a broad spectrum of applications. A series of triazine-based molecules, that is, 2,4,6-tri(9H-carbazol-9-yl)-1,3,5-triazine (TCZT), 2,4,6-tri(1H-indol-1-yl)-1,3,5-triazine (TIDT), and 2,4,6-tris(3,6-di-tert-butyl-9H-carbazol-9-yl)-1,3,5-triazine (TDBCZT), exhibiting violet-blue emission were synthesized via a catalyst-free aromatic nucleophilic substitution reaction. These compounds possess a non-planar and twisted structure with favorable charge-transfer characteristics, demonstrating excellent thermal stability (decomposition temperatures of 370 °C, 384 °C, and 230 °C, respectively). Cyclic voltammetry analysis, combined with time-dependent density functional theory (TD-DFT) calculations at the B3LYP/6-31G(d) level, offered detailed insights into their electronic structures and electrochemical properties. Optical properties were systematically characterized using Ultraviolet–visible (UV–Vis) absorption and photoluminescence (PL) spectroscopy. The compounds exhibited violet-blue luminescence with emission peaks located at 397 nm, 383 nm, and 402 nm in toluene, respectively. In their respective films, the compounds exhibited varying degrees of spectral shifts, with emission peaks at 408 nm, 381 nm, and 369 nm. Moreover, the CIE (Commission Internationale de l’Éclairage) coordinates of TIDT in toluene were (0.155, 0.067), indicative of excellent violet purity. These compounds demonstrated significant two-photon absorption (TPA) properties, with cross-sections of 4.6 GM, 15.3 GM, and 7.4 GM, respectively. Notably, they exhibited large molar absorptivities and substantial photoluminescence quantum yields (PLQYs), suggesting their potential for practical applications as violet-blue fluorescent materials.

## 1. Introduction

The advancement of highly efficient and economical violet-blue fluorescent materials is essential for enabling a broad spectrum of applications, such as displays with full color [1,2,3], devices for storing information at high density [4,5], and non-antibacterial therapeutic approaches [6]. Compared to inorganic violet-blue-emitting materials, organic violet-blue-emitting ones exhibit advantages such as flexibility [7,8], self-emissive characteristics [9], superior color purity [10], and enhanced efficiency [11]. Among them, D-A-type organic violet-blue-emitting materials are particularly significant as they can expand the exciton recombination region, impart bipolar transmission characteristics to the emitter, and enhance charge-transfer (CT) ability. These properties facilitate efficient electron transfer within the molecule, leading to superior luminescence performance [12,13,14]. However, inorganic violet-blue-emitting materials, especially Gallium Nitride (GaN) [15], continue to dominate the market owing to their high luminous efficiency [16], low cost [17], and excellent stability [18]. Organic violet-blue-emitting ones, on the other hand, remain limited due to their inherent wide bandgap characteristics [14]. The thermal stability of existing materials remains unsatisfactory, as most conventional emitters possess low decomposition temperatures, rendering them incompatible with high-temperature vacuum deposition processes [19]. Additionally, the synthesis costs of these materials remain excessively high owing to multi-step reactions that necessitate precious metal catalysts and intricate purification processes [20]. Moreover, efficiency roll-off continues to be a pivotal challenge, predominantly attributed to triplet-triplet annihilation and exciton quenching at elevated current densities [21]. Color purity limitations also remain unresolved, as many materials are unable to achieve the necessary narrow emission bandwidth and CIE y-coordinates below 0.08 for violet-blue applications [22]. Consequently, achieving high-performance violet-blue-emitting materials remains a substantial challenge within the domain of organic optoelectronics.

**Scheme 1:**
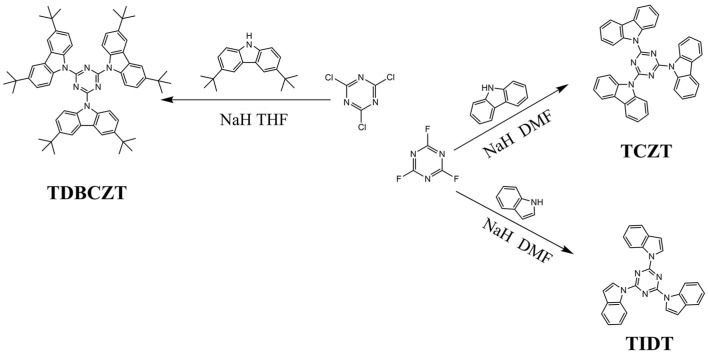
Molecular architectures of TCZT, TIDT, and TDBCZT, along with their synthesis via a catalyst-free aromatic nucleophilic substitution reaction.

This research presents the syntheses and comprehensive characterization of a set of D-A triazine-based molecules exhibiting violet-blue emission, specifically TCZT, TIDT, and TDBCZT (Scheme 1). Notably, the synthesis and characterization methods employed herein differ significantly from conventional approaches [23,24]. Specifically, the target compounds were obtained via a catalyst-free aromatic nucleophilic substitution reaction, as illustrated in Scheme 1. This one-step synthesis method yielded all compounds with high efficiency and ensured that the final products met the desired functional requirements.

## 2. Materials and Methods

### 2.1. Materials and Instruments

All solvents and chemical reagents utilized in this research were procured commercially and employed directly without any specific pretreatment unless otherwise stated. ^1^H NMR and ^13^C NMR spectra were obtained using a Bruker Advance-400 MHz spectrometer (Bruker, Billerica, MA, USA). High-resolution mass spectra were acquired using an Agilent Q-TOF 6510 mass spectrometer (Agilent Technologies Inc., Santa Clara, CA, USA). A Mettler Toledo thermogravimetric analysis (TGA)/differential scanning calorimetry (DSC) analyzer (Mettler-Toledo International Trade (Shanghai) Co., Ltd., Shanghai, China) was used to collect TGA and DSC data, conducted under N_2_ with a heating rate of 10 °C per minute, starting from room temperature. The electrochemical performance of the compounds, as evaluated through cyclic voltammetry, was assessed using the CHI660D electrochemical analyzer supplied by Shanghai Chenhua Instrument Co., Ltd., Shanghai, China. A three-electrode configuration was utilized, consisting of a glassy carbon electrode as the working electrode, a platinum electrode serving as the auxiliary electrode, and a Ag/AgCl electrode acting as the reference electrode. The electrolyte employed consisted of a 0.1 M solution of tetrabutylammonium perchlorate dissolved in dichloromethane (DCM). The EHOMO value was subsequently calculated by utilizing the equation *E*_HOMO_ = −e(*E*_onset(ox)_ − *E*_1/2,FOC_) – 4.8 eV [25]. UV–Vis absorption spectroscopy was conducted using a Hitachi U-2910 spectrophotometer (Hitachi Company, Tokyo, Japan). PL spectra were acquired with a F-2700 fluorescence spectrophotometer (Hitachi). The films were fabricated as follows: Prepare a THF solution with a concentration of 1 × 10^−4^ mol/L. Spin-coat it on a quartz sheet at a speed of 3000 rpm for 30 s, and then vacuum dry it at 50 °C for 4 h. The morphological characteristics of the thin film samples were analyzed using scanning electron microscopy (SEM; model S-4800, manufactured by Hitachi). To improve surface conductivity for clearer imaging, the samples were sputter-coated with a thin layer of gold. SEM images were captured under an accelerating voltage of 5.0 kV and a working distance of 12.6 mm. Photoluminescence quantum yields (PLQYs) were determined by referencing quinine sulfate in a 0.1 M H_2_SO_4_ solution [26]. The absolute quantum yields were measured with a Edinburgh FLS980 spectrometer from Edinburgh Instruments Ltd., (Livingston, UK) using an integrating sphere setup. Theoretical calculation was performed by utilizing the Gaussian 16 software package. The DFT approach was employed to optimize the molecular geometry at the B3LYP/6-31G(d)/PCM level in toluene. The vertical electron transition was obtained at the B3LYP/6-31G(d) level using TD-DFT method. The two-photon excited fluorescence (TPEF) was detected with a SpectroPro300i spectrometer. The excitation source for the TPEF measurements covered wavelengths ranging from 690 to 860 nm at room temperature. Solutions of TCZT, TIDT, and TDBCZT were prepared in tetrahydrofuran (THF) at a concentration of 10^−4^ M, while a 10^−4^ M methanol solution of coumarin 307 served as the reference standard (Figure S14). TPA cross-sections were determined using the two-photon induction method, with the cross-sections calculated according to Equation (1) [27]. Notably, *r* and *s* denote the reference and sample, respectively. δ represents the TPA cross-section, Φ signifies the fluorescence quantum yield, *c* indicates the concentration of the solution, *n* denotes the solution’s refractive index, and *F* corresponds to the integral intensity of excited fluorescence.

(1) $$\delta_{s}=\delta_{r}\left(\frac{\Phi_{r}}{\Phi_{s}}\right)\left(\frac{c_{r}}{c_{s}}\right)\left(\frac{n_{r}}{n_{s}}\right)\left(\frac{\int F_{s}}{\int F_{r}}\right)$$

### 2.2. Synthesis and Characterization

All compounds were synthesized via a coupling reaction [28]. The preparation of these three compounds could be accomplished at moderate temperatures within a reasonable timeframe, while maintaining a safe and straightforward procedure. The supporting information (Figures S1–S9) includes the characterization data for the synthesized compounds, such as the ^1^H NMR, ^13^C NMR, and HRMS spectra of the synthesized compounds. The synthetic pathways are depicted in Scheme 1, and the specific synthesis procedures are outlined below:

2,4,6-Tri(9H-carbazol-9-yl)-1,3,5-triazine (TCZT). In a 350 mL round-bottom flask, sodium hydride (NaH, 0.48 g, 12 mmol) and carbazole (1.67 g, 10 mmol) were sequentially added under N_2_, followed by the introduction of anhydrous dimethylformamide (DMF, 40 mL). Subsequently, cyanuric fluoride (0.25 mL, 3 mmol) was carefully injected into the mixture of reactants. The resulting solution was agitated at room temperature for 8 h under a continuous N_2_ purge. Water (50 mL) was introduced to terminate the reaction. The resulting mixture was then extracted three times using CH_2_Cl_2_. The crude product was purified by means of column chromatography (eluent: petroleum ether/ethyl acetate = 20/1, *v*/*v*). The white solid (0.69 g, 40% yield) was successfully isolated. ^1^H NMR (400 MHz, CDCl_3_) δ 9.05–9.01 (m, 1H), 8.16–8.12 (m, 1H), 7.50–7.26 (m, 2H) ppm. ^13^C NMR (400 MHz, CDCl_3_), δ 164.33, 138.80, 127.14, 126.53, 123.52, 119.79, 117.54 ppm. HRMS (*m*/*z*): [M + H]^+^ calculated for (C_39_H_24_N_6_): 577.2141, found: 577.2125.

2,4,6-Tri(1H-indol-1-yl)-1,3,5-triazine (TIDT). In a 350 mL flask, NaH (0.48 g, 12 mmol) and indole (1.17 g, 10.0 mmol) were dissolved in anhydrous DMF (40 mL) under a nitrogen atmosphere. Subsequently, cyanuric fluoride (0.25 mL, 3 mmol) was introduced into the reaction mixture. The solution was stirred for 8 h at 40 °C under a continuous nitrogen purge. Upon completion of the reaction, it was extinguished by the addition of water (50 mL). The resultant mixture was extracted three times using CH_2_Cl_2_. The crude product was then purified by column chromatography (eluent: petroleum ether/ethyl acetate = 20/1, *v*/*v*), yielding a white solid (0.77 g, 53% yield.) ^1^H NMR (400 MHz, CDCl_3_), δ 8.85 (dt, J = 8.4, 0.9 Hz, 1H), 8.38 (d, J = 3.8 Hz, 1H), 7.68 (dt, J = 7.6, 1.0 Hz, 1H), 7.46 (ddd, J = 8.4, 7.1, 1.3 Hz, 1H), 7.34 (ddd, J = 8.0, 7.3, 1.0 Hz, 1H), 6.85 (dd, J = 3.8, 0.8 Hz, 1H) ppm. ^13^C NMR (400 MHz, CDCl_3_), δ 163.95, 135.90, 132.29, 126.23, 124.94, 123.80, 121.85,117.10, 109.63 ppm, HRMS (*m*/*z*): [M + H]^+^ calculated for (C_27_H_18_N_6_): 427.1671, found: 427.1669.

2,4,6-Tri(3,6-Di-tert-butylcarbazole-9-yl)-1,3,5-triazine (TDBCZT). In a 350 mL flask under nitrogen, NaH (0.6 g, 15 mmol) and 3,6-Di-tert-butylcarbazole (2.97 g, 10 mmol) were dissolved in anhydrous THF (50 mL). Cyanuric chloride (0.55 g, 3 mmol) was subsequently introduced. The resulting mixture was stirred at 60 °C for a duration of 10 h. To terminate the reaction, water (50 mL) was added, and the mixture was then extracted three times using CH_2_Cl_2_. The crude product obtained was further purified via column chromatography (eluent: petroleum ether), producing a white solid (0.82 g, yield 30%). ^1^H NMR (400 MHz, CDCl_3_), δ 8.89 (d, J = 8.9 Hz, 1H), 8.11 (d, J = 2.0 Hz, 1H), 7.51 (dd, J = 8.9, 2.1 Hz, 1H), 1.50 (s, 9H) ppm. ^13^C NMR (400 MHz, CDCl_3_), δ 164.14, 146.35, 137.21, 126.52, 124.51, 117.18, 115.79, 34.86, 31.86 ppm. HRMS (m/z): [M + H]^+^ calculated for (C_63_H_72_N_6_): 913.5897, found: 913.5858.

## 3. Results and Discussion

### 3.1. Thermal Properties

**Figure 1:**
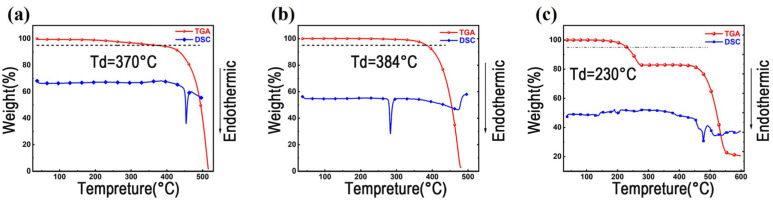
TGA and DSC curves of (**a**) TCZT, (**b**) TIDT, and (**c**) TDBCZT, the dash lines indicating the compounds with a 5% weight loss.

TCZT, TIDT, and TDBCZT are a type of fluorescent material that exhibits comprehensive performance. Their excellent thermal properties enable them to be utilized at both high and low temperatures for extended periods of time. The thermal characteristics of these materials were investigated using simultaneous DSC and TGA in an atmosphere of nitrogen. The melting point of TIDT was observed to be 290 °C. No distinct melting point was detected for TCZT and TDBCZT. According to the TGA plots presented in Figure 1, the temperatures at which decomposition occurs (*T*_d_), resulting in a 5% weight loss, were identified as follows: TCZT exhibited a *T*_d_ of 370 °C, TIDT showed a *T*_d_ of 384 °C, and TDBCZT displayed a lower *T*_d_ value of 230 °C. The thermal decomposition temperatures of TCZT and TIDT were found to be significantly high, nearly equivalent to those of the 1,3,5-triazine derivatives DPA-XA-TRZ and DPA-FR-TRZ, which were measured at 404 °C and 372 °C, respectively [29]. The statement also indicates the stability of TCZT and TIDT in withstanding thermal decomposition during the vacuum deposition process [30]. The tert-butyl substituent, in combination with carbazole, predominantly contributes to the lowest *T*_d_ observed for TDBCZT [31]. Specifically, TDBCZT experiences a mass loss of approximately 16.6% during the initial stage of decomposition, which corresponds to roughly half of the weight of its tert-butyl group.

### 3.2. Electrochemical Properties 

**Figure 2:**
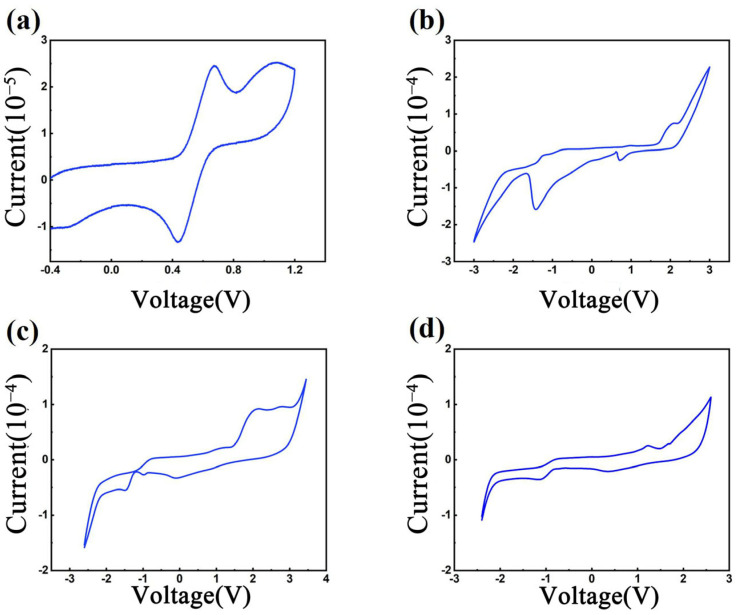
Cyclic voltammograms for (**a**) Fc/Fc^+^, (**b**) TCZT, (**c**) TIDT, and (**d**) TDBCZT.

Cyclic voltammetry (CV) was employed to characterize the electrochemical properties of the compounds. Figure 2a showed the CV curve of Fc/Fc^+^ system. The half-wave potential of Fc/Fc^+^ (E_1/2_,FOC) was determined to adjust the reference electrode for calibration purposes [32], and the value of *E*_1/2_,FOC was 0.56 V. Figure 2b–d showed the CV curves of TCZT, TIDT, and TDBCZT. The oxidation peak for TCZT occurred at 2.09 V, with an onset oxidation potential of 1.42 V. In contrast, TIDT exhibited an oxidation peak at 2.15 V and an onset oxidation potential of 1.67 V. The reduction peak for TCZT was observed at −1.42 V, whereas the peak for TIDT was identified at −1.50 V. Therefore, both of them were irreversible redox processes [33]. Different from the previous two, the oxidation peak for TDBCZT occurred at 1.23 V, with an onset oxidation potential of 0.67 V, while the reduction peak was observed at −1.17 V. Therefore, the redox process of TDBCZT was deemed to exhibit quasi-reversible characteristics. The HOMO energy levels of TCZT, TIDT, and TDBCZT were calculated as −5.67 eV, −5.92 eV, and −4.92 eV, respectively. The LUMO energy levels were calculated based on their optical band gaps and were determined to be −2.49. eV, −2.27 eV, and −2.11 eV, respectively [34].

### 3.3. Photophysical Properties and Theoretical Calculations

**Figure 3:**
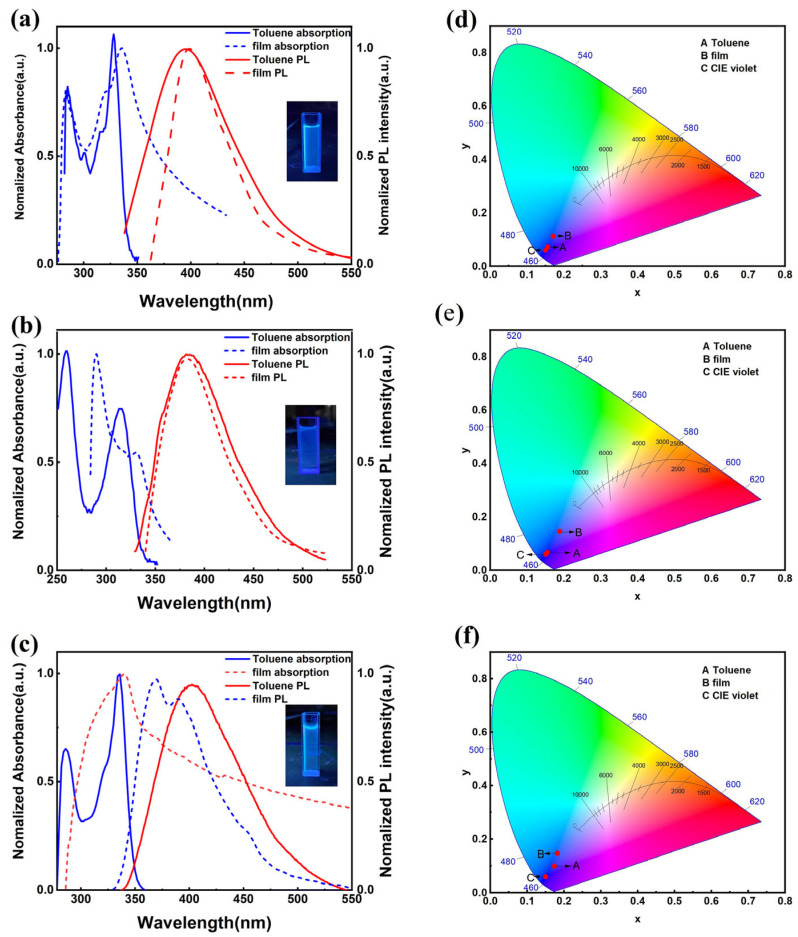
Normalized UV–Vis absorption and PL spectra of (**a**) TCZT, (**b**) TIDT, and (**c**) TDBCZT in toluene and films. The CIE1931 coordinates of (**d**) TCZT, (**e**) TIDT, and (**f**) TDBCZT. The insets in the middle-right section of figures (**a**–**c**) illustrating the fluorescence emission of the corresponding compounds (at a concentration of 10^−6^ M in toluene) inside a quartz cuvette when exposed to irradiation from a fluorescent lamp.

Essential photophysical information were shown in Table 1. Figure 3a–c shows the UV–Vis absorption and PL spectra of TCZT, TIDT, and TDBCZT in toluene and neat films, respectively. Distinct absorption peaks were detected at wavelengths of 285 nm and 328 nm for TCZT, 260 nm and 317 nm for TIDT, and 286 nm and 336 nm for TDBCZT in toluene. These absorption bands can be attributed to the π-π* transitions originating from the donor units [35]. Specifically, the absorption band of TIDT exhibits a broader profile compared to TCZT and TDBCZT in toluene. This can be attributed to the twisted non-planar conformation induced by indole substituents on the triazine core, which enhances vibrational coupling between ground and excited states. The strong electron-donating nature of indole groups intensifies intramolecular charge transfer (ICT) from the aryl donors to the triazine acceptor, resulting in multiple overlapping electronic transitions across the 300–350 nm range [36,37,38]. While the absorption band of TDBCZT in neat films is higher, which can be attributed to the bulky tert-butyl groups on carbazole units disrupting close π-π stacking, leading to enhanced vibrational coupling and static disorder in the solid state [39]. This inhomogeneous environment leads to a broadened absorption profile by distributing ICT transition energies.

The compounds exhibited violet-blue luminescence in toluene, with maximum emission peaks at 397 nm, 383 nm, and 402 nm, associated with Full Width at Half Maximum (FWHM) values of 93 nm, 80 nm, and 92 nm, respectively. In contrast, the fluorescence peaks of all the compound films have a very small shift or a significant blue shift. Notably, the neat film emission peak of TDBCZT was observed at 369 nm, indicating a significant blue shift compared to its solution state in toluene. This phenomenon can be attributed to the loose molecular packing, which results in weakened π-π* transitions and an increased energy gap. The weakened intermolecular interactions strengthen the dominance of intramolecular conjugation, leading to an increase in the HOMO-LUMO energy gap. Moreover, the suppressed intermolecular charge transfer in the solid state further contributes to the blue-shifted emission by reducing the stabilization of the excited state [40]. In addition, significant self-quenching was observed in these materials, as evidenced by the sharp decline in PLQYs of TCZT, TIDT, and TDBCZT when transitioning from toluene solution to the neat film state. This phenomenon can be attributed to the self-absorption effect in the aggregated state, where emitted photons are reabsorbed due to the strong spectral overlap between the absorption (300–400 nm) and emission (369 nm) bands. Such overlap enhances non-radiative decay pathways, leading to the observed emission quenching [41,42].

As shown in Figure 3d–f, the CIE1931 coordinates for TCZT, TIDT, and TDBCZT in toluene are (0.155, 0.072), (0.155, 0.067), and (0.173, 0.095), respectively. Notably, TCZT and TIDT exhibit superior color purity in toluene relative to the standard violet CIE coordinate of (0.150, 0.060). These results are also comparable to those of TIC-BO, measured at (0.160, 0.050) [43], and DMACN-B, measured at (0.151, 0.045) [44]. With the high thermal stability (*T*_d_ = 384 °C) and narrow emission spectrum (CIEy = 0.067) of TIDT, this material holds unique advantages in deep-blue OLEDs requiring high-temperature processing techniques. It effectively avoids the degradation of device lifetime caused by thermal decomposition in commercial materials, while meeting the stringent color purity requirements of Rec.2020 for wide-gamut displays. Furthermore, it was observed that the PLQYs of all compounds exhibited higher values in low-polarity solvents. Specifically, the PLQYs for TCZT, TIDT, and TDBCZT varied within the ranges of 20.6% to 46.0%, 7.4% to 20.0%, and 20.8% to 47.4% in various solvents, respectively (Tables S1–S3). In addition, the PLQYs of TCZT, TIDT, and TDBCZT in films were found to be 1.43%, 2.67%, and 3.16%, respectively. Notably, the PLQYs of TCZT and TDBCZT in solutions were higher than those of TIDT. This indicates that the efficiency of intramolecular charge transfer (ICT) between the donor and acceptor components in TIDT is more pronounced compared to TCZT and TDBCZT. The enhanced ICT in TIDT can be attributed to its strong electron-donating indole group, extended conjugation system. Consequently, this efficient electron transfer may lead to non-radiative transitions during the ICT process, thereby reducing the PLQYs [45,46]. Notably, TCZT, TIDT, and TDBCZT emit violet-blue fluorescence, making them promising candidates as light-emitting layers in violet-blue OLEDs and as potential fluorescence probes.

The UV–Vis absorption and PL spectra of TCZT, TIDT, and TDBCZT in various solvents are presented in Figure S10, while the photophysical parameters are summarized in Tables S1–S3. As illustrated in Figure S10 and Figure 3, these compounds displayed comparable absorption characteristics across various solvents as well as in films, with minimal and insignificant peak shifts. This suggests that the ground states were largely unaffected by the dipole moment. Additionally, this observation indicates that the absorption behavior of these compounds in dilute solutions is predominantly governed by π-π* transitions, which are typically less susceptible to the influence of polar solvents in aromatic compounds [47]. The emission peaks of all compounds were observed within the range of wavelengths 371 to 450 nm. Each of the three compounds exhibited distinct solvatochromic shifts, which were accompanied by an increase in their FWHM. Specifically, the emission peak of TDBCZT red-shifted approximately 80 nm from hexane to acetonitrile. This phenomenon can be attributed to the delocalized conjugation of the triazine ring [48] and the occurrence of charge transfer (CT) during photoexcitation [49]. Specifically, the aromatic nature of the triazine ring and its π-conjugation with carbazole moieties decrease the energy difference between the ground state and the excited state. Polar solvents can further stabilize the charge-transfer (CT) state in the excited state via dipole–dipole interactions or hydrogen bonding, resulting in a shift towards longer wavelengths in the emission spectrum.

**Figure 4:**
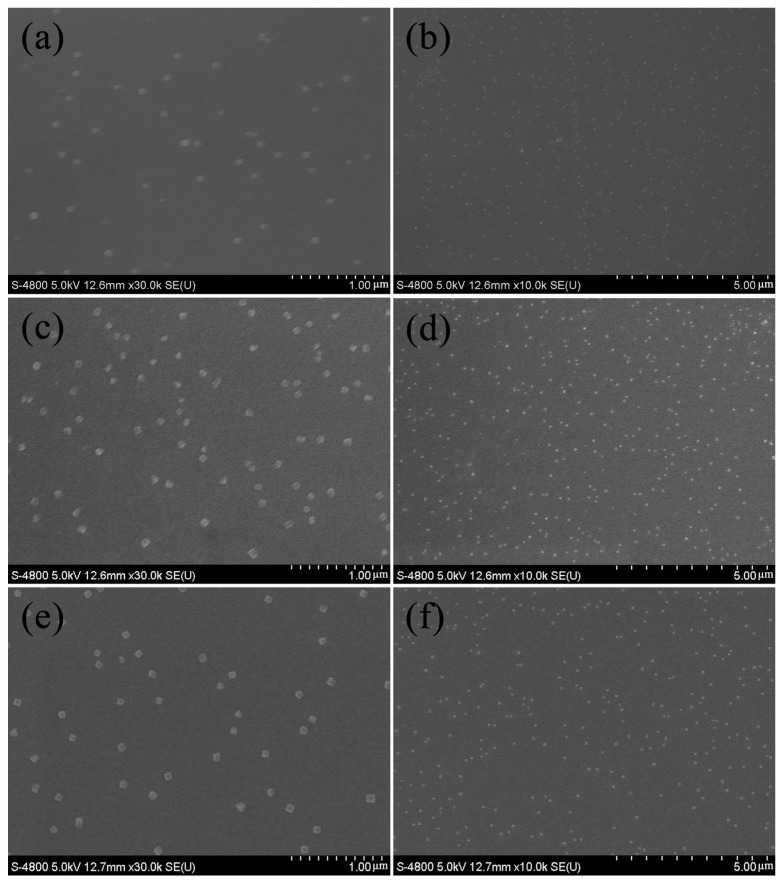
SEM micrograph with micron scale bar = 1.5 μm of (**a**,**b**) TCZT, (**c**,**d**) TIDT, and (**e**,**f**) TDBCZT in films.

As shown in Figure 4, scanning electron microscopy (SEM) was employed to characterize the microstructure of the neat films. It can be seen from the figure that the nanoparticles formed by the molecules are uniform in size and the estimated average grain sizes were determined to be 70–110 nm for TCZT, 80–100 nm for TIDT, and 90–100 nm for TDBCZT, respectively. All three films exhibited a relatively uniform grain distribution, with notable differences in particle packing density: TDBCZT exhibited the most loosely packed particle distribution, likely due to the steric hindrance effect caused by its tert-butyl groups [50], whereas TIDT presented the most densely packed microstructure. The lower PLQYs observed in the neat films, as compared to those in toluene, can be ascribed to the combined effects of self-absorption and fluorescence quenching behavior in the neat film [51], which diminish the photoluminescence efficiency.

**Figure 5:**
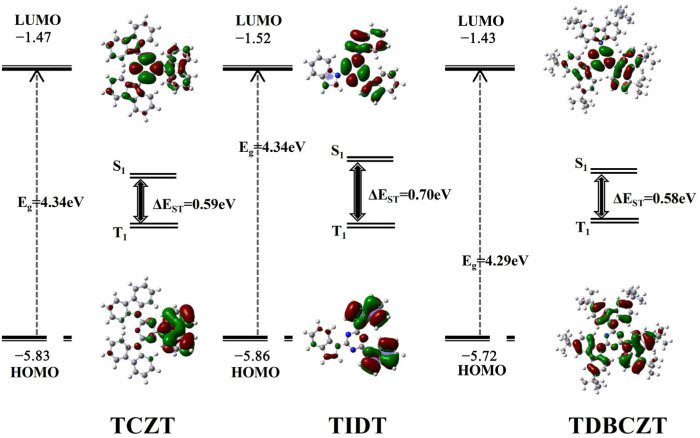
Molecular orbitals and energy diagrams using DFT for TCZT, TIDT, and TDBCZT.

In order to attain a deeper and more extensive comprehension of the frontier molecular orbitals (refer to Figure 5 and Figures S11–S13) and absorption characteristics, we performed theoretical calculations utilizing the Gaussian 16 software package [52]. The molecular geometry was optimized via DFT methods at the B3LYP/6-31G(d) level with a polarizable continuum model (PCM) for toluene solvent effects. The vertical electron transition was determined at the B3LYP/6-31G(d) theoretical level employing the TD-DFT method. The electron density of the highest occupied molecular orbitals (HOMO) in compounds TCZT and TIDT is predominantly localized on specific regions of the electron donor moieties, whereas in compound TDBCZT, the HOMO electron density is more extensively distributed across nearly all electron donor sites. On the other hand, the lowest unoccupied molecular orbitals (LUMOs) display electron density predominantly localized over the triazine unit and portions of the electron-donating moieties in these compounds. Theoretical calculations of the HOMO energy levels for TCZT and TIDT are in close agreement with those derived from cyclic voltammetry (CV) measurements. In contrast, the HOMO energy level of TDBCZT is marginally elevated compared to the CV results. These compounds exhibit markedly elevated Δ*E*_ST_ values of 0.59 eV, 0.70 eV, and 0.58 eV in comparison to DtCzB-DPTRZ, DtCzB-TPTRZ, DtCzB-PPm, and DtCzB-CNPm, which exhibit the Δ*E*_ST_ values of 0.18 eV, 0.11 eV, 0.11 eV, and 0.15 eV, respectively [53]. This disparity in Δ*E*_ST_ hinders the reverse intersystem crossing (RISC) process. As a result, these compounds are unsuitable for application as materials exhibiting thermally activated delayed fluorescence (TADF).

**Figure 6:**
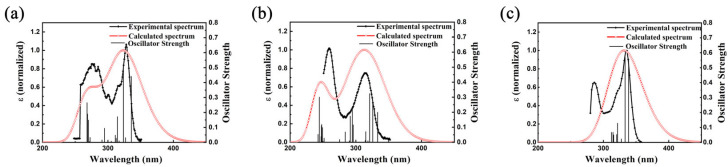
Theoretical UV–vis absorption spectra investigated employing the TD-DFT/B3LYP/6-31 g(d) method for (**a**) TCZT, (**b**) TIDT, and (**c**) TDBCZT in contrast to the experimental UV–Vis absorption spectra of TCZT, TIDT, and TDBCZT in toluene.

Figure 6 illustrates that the UV–Vis absorption spectra computed through TD-DFT show excellent consistency with the experimentally obtained values in toluene. The maximum absorption peaks for TCZT, TIDT, and TDBCZT in toluene were noticed at 328 nm, 317 nm, and 336 nm, respectively. These peaks can be attributed to electronic transitions from HOMO to LUMO + 1 and HOMO-3→LUMO for TCZT, HOMO-2→LUMO and HOMO→LUMO + 2 for TIDT, and HOMO→LUMO, HOMO-2→LUMO + 1, HOMO-1→LUMO for TDBCZT (Tables S4–S6).

**Figure 7:**
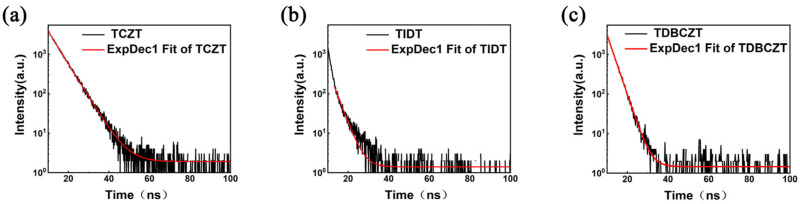
Lifetime decay curve of (**a**) TCZT, (**b**) TIDT, and (**c**) TDBCZT.

Figure 7 illustrates the fluorescence decay lifetimes of the three compounds in toluene, all of which displayed a fluorescence decay process with a single-exponential characteristic in the nanosecond range lifetimes of 5.21 ns, 3.52 ns, and 2.87 ns, respectively, without any delayed fluorescence. These lifetimes are notably shorter compared to those reported by Zhang et al. for an exciplex-forming host system, where the prompt fluorescence lifetime was 17.4 ns and the delayed fluorescence lifetime was 2.8 µs [54]. Consequently, these compounds do not possess the characteristics of thermally activated delayed fluorescence. The conclusion that these three compounds are not TADF materials is supported by the large Δ*E*_ST_ values obtained from theoretical calculations [55].

**Figure 8:**
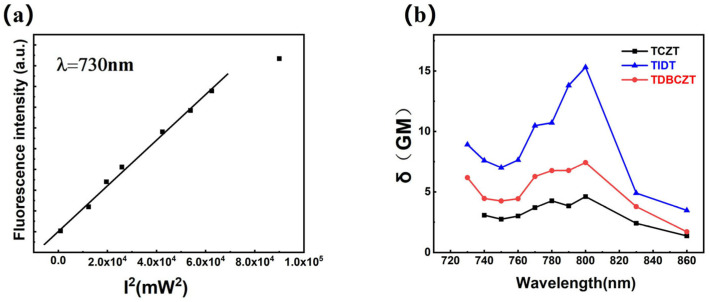
(**a**) The quadratic power dependence of two-photon-excited fluorescence for TCZT when the excitation wavelength is set to 730 nm. (**b**) δ values of TCZT, TIDT, and TDBCZT at different excitation wavelengths.

The TPA properties of star-shaped triazine derivatives have scarcely been investigated in prior studies. In this work, the TPA characteristics of three compounds were evaluated using a 1 × 10^−4^ mol/L solution of coumarin 307 in methanol as the reference standard for calculating the TPA cross-section (δ). The TPEF spectra of coumarin 307, TCZT, TIDT, and TDBCZT at various excitation wavelengths are presented in Figure S14. To ensure that all detected fluorescence was solely derived from two-photon excitation, we examined the quadratic power dependence of TCZT’s two-photon excitation fluorescence at 730 nm. As illustrated in Figure 8a, when the laser power was below 250 mW, the intensity of the two-photon-excited fluorescence exhibited a quadratic relationship with the incident energy, confirming that the emitted signal arises from a two-photon absorption process. Consequently, the laser power was adjusted to 235 mW during the measurements.

The experimental results demonstrated that these compounds exhibited both TPA and TPEF values within the excitation wavelength range of 730–860 nm. The highest *δ* values for TCZT, TIDT, and TDBCZT were investigated to be 4.6, 15.3, and 7.4 GM, respectively, at a stimulation wavelength of 800 nm (Figure 8b). This indicates that TIDT exhibits a superior response capability under two-photon excitation compared to the other compounds. The favorable TPA properties of these three compounds can be attributed to their conjugated systems and branch chain lengths, which feature relatively strong electron-accepting triazine groups [56,57].

## 4. Conclusions

In this study, three organic small molecules TCZT, TIDT, and TDBCZT were successfully synthesized via a catalyst-free aromatic nucleophilic substitution reaction. Subsequently, their thermal, electrochemical, and optical properties were thoroughly investigated and analyzed using theoretical calculations. The compounds exhibited excellent thermal stability, featuring decomposition temperatures (*T*_d_) of 370 °C, 384 °C, and 230 °C. TCZT, TIDT, and TDBCZT exhibited violet-blue luminescence in toluene with the highest emission peaks at 397 nm, 383 nm, and 402 nm, respectively. The corresponding CIE coordinates for them were (0.155, 0.072), (0.155, 0.067), and (0.153, 0.065). In contrast, the film samples displayed peak emissions at 408 nm, 381 nm, and 369 nm, indicating spectral shifts. The PLQYs of TCZT and TIDT in toluene and TDBCZT in hexane were 46.0%, 18.0%, and 47.4%, respectively, classifying them as highly efficient violet-blue fluorescent materials. The PLQYs of their corresponding compound films were 1.43%, 2.67%, and 3.16%. The maximum δ value of TIDT was measured at 15.3 GM, which signifies its notable TPA properties among 1,3,5-triazine-based dyes. Consequently, these triazine-based D-A molecules exhibit potential applications in violet-blue OLEDs, fluorescence probes, and other relevant fields.

## Figures and Tables

**Table 1:** Selected optical properties for TCZT, TIDT, and TDBCZT.

Compounds	λ_abs_^(a)^ [nm]	λ_em_^(b)^ [nm]	FWHM ^(c)^ [nm]	CIE (x, y) ^(d)^	Фf ^(e)^ [%]	δmax ^(f)^ [GM]
TCZT	285, 328/284, 336	397/408	93/61	(0.155, 0.072)	46.0/1.43	4.6
TIDT	260, 317/291, 331	383/381	80/75	(0.155, 0.067)	18.0/2.67	15.3
TDBCZT	286, 336/288, 340	402/369	92/79	(0.173, 0.995)	31.4/3.16	7.4

^(a)^ Absorption peaks in toluene solution (10^−6^ M) and in the neat film state, respectively. ^(b)^ Emission peaks in toluene solution (10^−6^ M) and in the neat film state, respectively. ^(c)^ FWHM in toluene solution (10^−6^ M) and in the neat film state, respectively. ^(d)^ CIE coordinates in toluene solution (10^−6^ M). ^(e)^ Fluorescence quantum yields at the peak absorption wavelengths in toluene were determined by utilizing quinine sulfate in 0.1 M H_2_SO_4_ solution (Ф = 0.55) as a reference and absolute fluorescence quantum yields measured in neat film state. ^(f)^ TPA cross-section (δ) in tetrahydrofuran solution (10^−5^ M) at 800 nm.

## Data Availability

The original contributions introduced in this research are comprehensively documented within the paper and additional Supplementary Materials. Any further inquiries should be directed to the author responsible for correspondence.

## Supplementary Materials

The following supporting information can be downloaded at https://www.mdpi.com/article/10.3390/ma18092043/s1. Figures S1–S9: ^1^H and ^13^C NMR and HRMS spectra of TCZT, TIDT, and TDBCZT; Figure S10: Normalized UV–Vis absorption and PL spectra of TCZT, TIDT, and TDBCZT; Figures S11–S13: Frontier molecular orbitals associated with the electronic transitions of TCZT, TIDT, and TDBCZT; Figure S14: The TPEF spectra of TCZT, TIDT, and TDBCZT at different excitation wavelength; Tables S1–S3: The photophysical properties of (a) TCZT, (b) TIDT, and (c) TDBCZT in various solvents; Tables S4–S6: Absorption wavelength and oscillator strength of TCZT, TIDT, and TDBCZT.

## Abbreviations

The abbreviations listed below are utilized within this manuscript:

D-A type	Donor–acceptor type
TD-DFT	Time-dependent density functional theory
DFT	Density functional theory
PL	Photoluminescence
PLQYs	Photoluminescence quantum yields
CT	Charge transfer
ICT	Intramolecular charge transfer
RISC	Reverse intersystem crossing
CV	Cyclic voltammetry
HRMS	High-resolution mass spectra
TGA	Thermogravimetric analysis
DSC	Differential scanning calorimetry
TPEF	Two-photon excited fluorescence
TPA	Two-photon absorption
FWHM	Full Width at Half Maximum
HOMO	Highest occupied molecular orbital
LUMO	Lowest occupied molecular orbital
TADF	Thermally activated delayed fluorescence
DCM	Dichloromethane
THF	Tetrahydrofuran
DMF	Dimethylformamide
