# Supplementary material for: Preparation, Thermal, and Optical Properties of D-A-Type Molecules Based on 1,3,5-Triazine for Violet-Blue Fluorescent Materials
Source: Materials (Basel). 2025 Apr 29;18(9):2043. doi: 10.3390/ma18092043 (PMC12072454; doi:10.3390/ma18092043)
Supplement: Supplementary file 1 [file materials-18-02043-s001.zip › materials-3554616-supplementary.pdf]

Electronic Supplementary Materials

Preparation, Thermal, and Optical Properties of D-A-Type Molecules Based on 1,3,5-Triazine for Violet-Blue Fluorescent Materials

Lu Wang [1,†], Enwang Du [2,†], Zhi Liu [1,*] and Zhiqiang Liu [1]

[1] State Key Laboratory of Crystal Materials and Institute of Crystal Materials, Shandong University, Jinan 250100, China
[2] School of Physics, Shandong University, Jinan 250100, China
* Correspondence: lz@sdu.edu.cn
† These authors made equal contributions to this work.

Contents:

Figure S1. ^{1}H NMR spectrum of TCZT in $CDCl_3$. 2
Figure S2. ^{13}C NMR spectrum of f TCZT in $CDCl_3$. 2
Figure S3. HRMS spectrum of TCZT in DCM. 3
Figure S4. ^{1}H NMR spectrum of TIDT in $CDCl_3$.......... 3
Figure S5. ^{13}C NMR spectrum of f TIDT in $CDCl_3$. 4
Figure S6. HRMS spectrum of TIDT in DCM. 4
Figure S7. ^{1}H NMR spectrum of TDBCZT in $CDCl_3$. 5
Figure S8. ^{13}CNMR spectrum of TDBCZT in $CDCl_3$. 5
Figure S9. HRMS spectrum of TDBCZT in $CDCl_3$. 6
Figure S10. Normalized absorption and PL spectra of (a) TCZT, (b) TIDT and (c) TDBCZT in various solvents. 6
Figure S11. Frontier molecular orbitals associated with the electronic transitions of TCZT. 7
Figure S12. Frontier molecular orbitals associated with the electronic transitions of TIDT. 7
Figure S13. Frontier molecular orbitals associated with the electronic transitions of TDBCZT.......... 8
Figure S14. The TPEF spectra of (a) coumarin 307 (b) TCZT (c) TIDT (d) TDBCZT at various excitation wavelengths. 8
Table S1. The photophysical data of TCZT in various solvents. 9
Table S2. The photophysical data of TIDT in various solvents. 9
Table S3. The photophysical data of TDBCZT in various solvents.......... 9
Table S4. Absorption wavelength and oscillator strength of TCZT evaluated by TD-DFT at the B3LYP/6-31G(d) 10
Table S5. Absorption wavelength and oscillator strength of TIDT evaluated by TD-DFT at the B3LYP/6-31G(d) 11
Table S6. Absorption wavelength and oscillator strength of TDBCZT evaluated by TD-DFT at the B3LYP/6-31G(d) 12

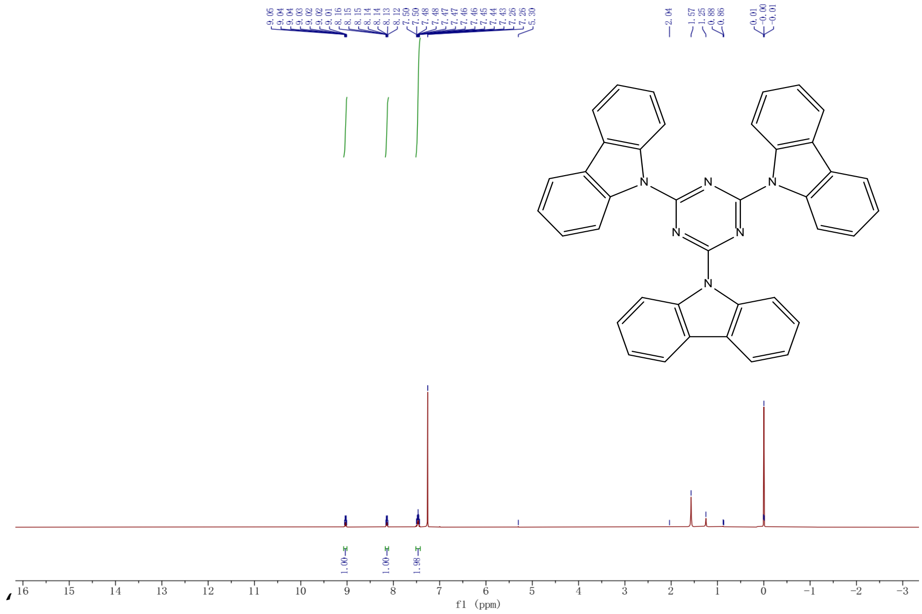

Figure S1. ^{1}H NMR spectrum of **TCZT** in $CDCl_3$.

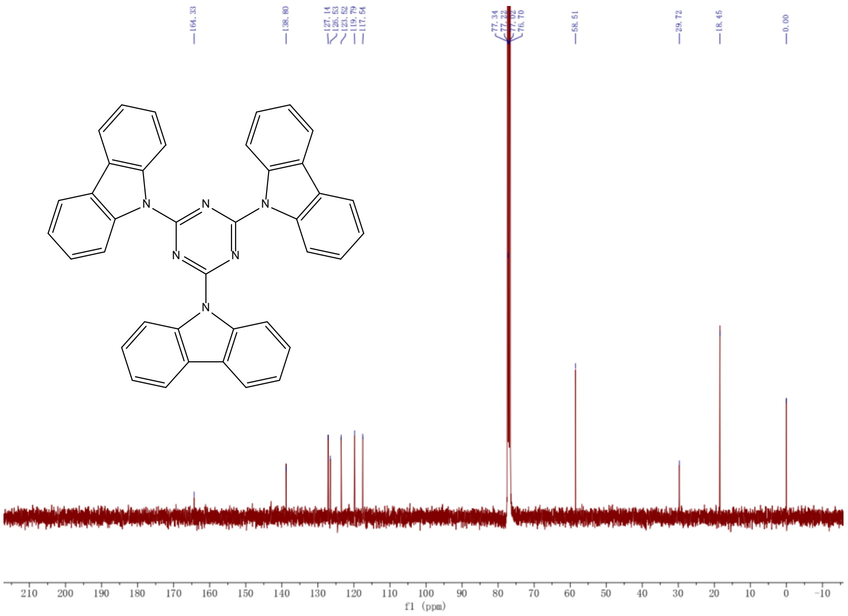

Figure S2. ^{13}C NMR spectrum of f **TCZT** in $CDCl_3$.

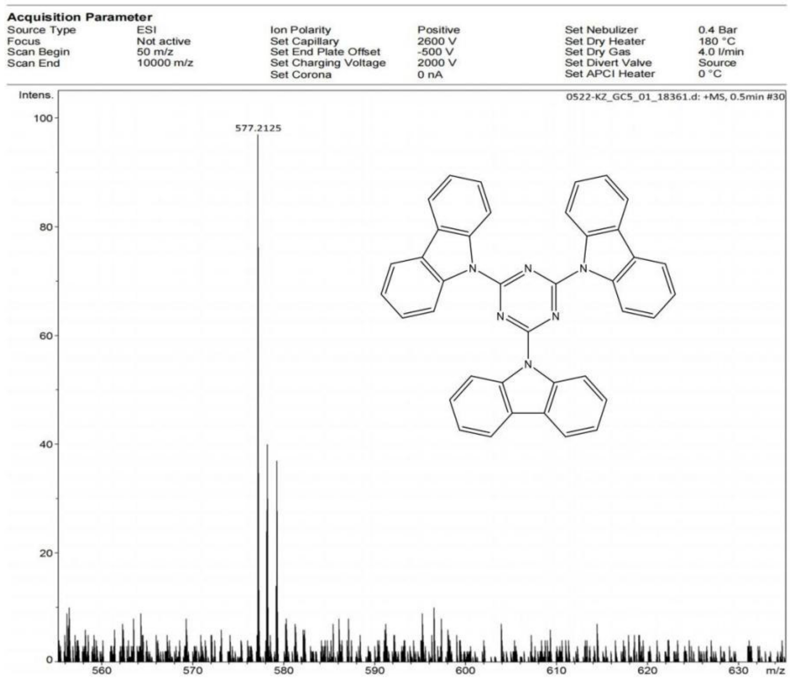

Figure S3. HRMS spectrum of **TCZT** in DCM.

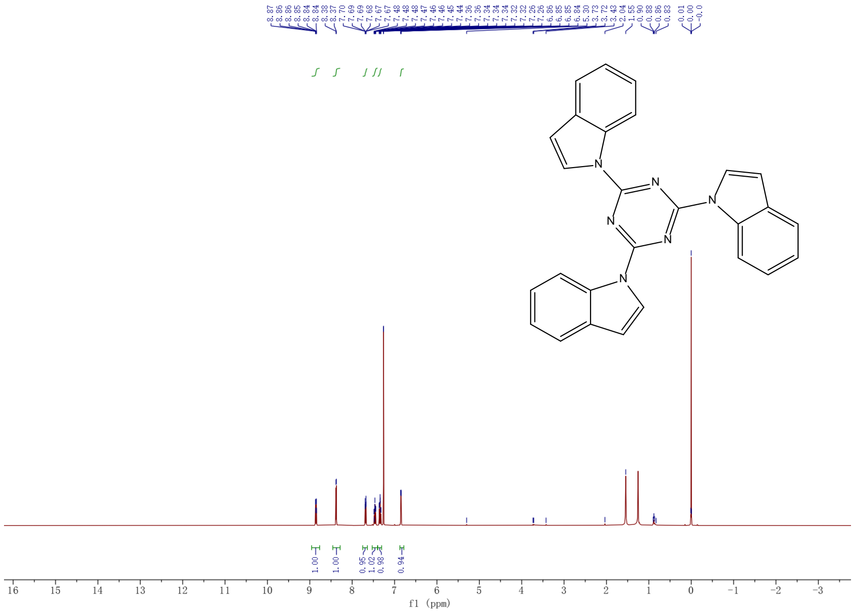

Figure S4. ^{1}H NMR spectrum of **TIDT** in $CDCl_3$.

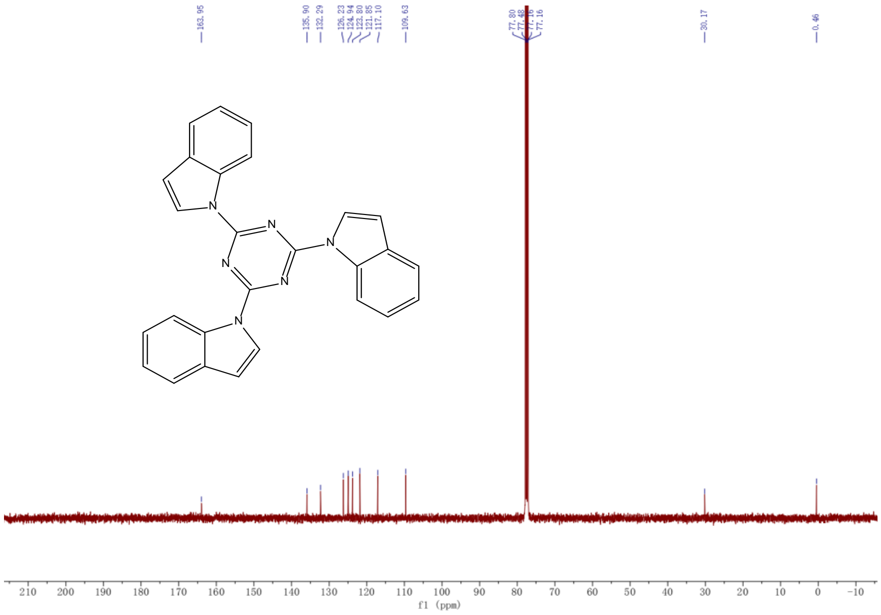

Figure S5. ^{13}C NMR spectrum of f **TIDT** in $CDCl_3$.

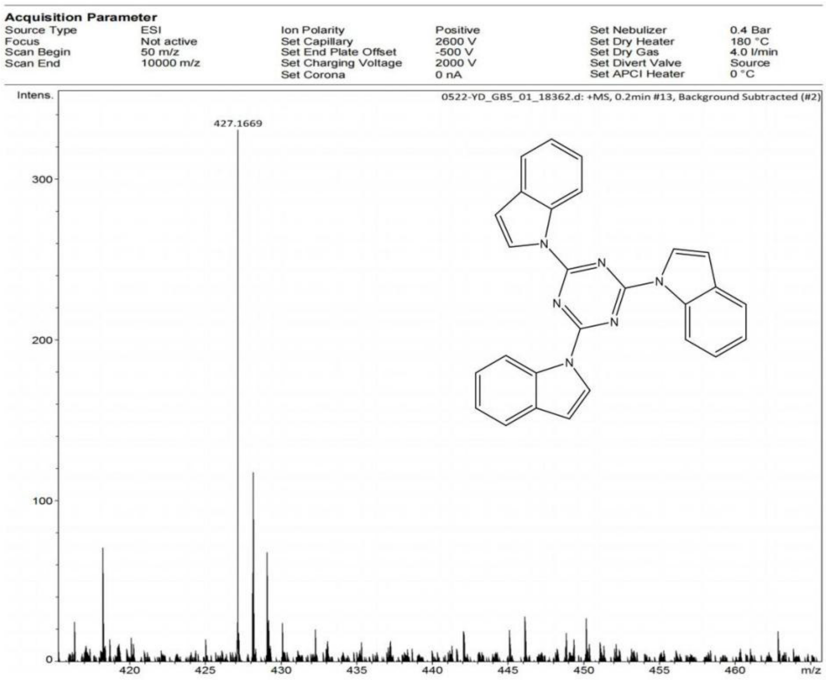

Figure S6. HRMS spectrum of **TIDT** in DCM.

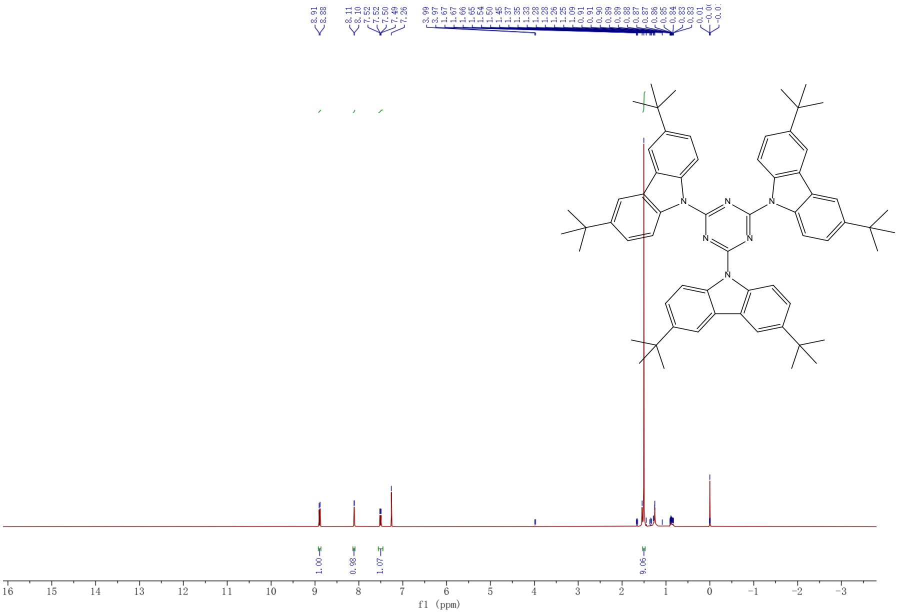

Figure S7. ^{1}H NMR spectrum of **TDBCZT** in $CDCl_3$.

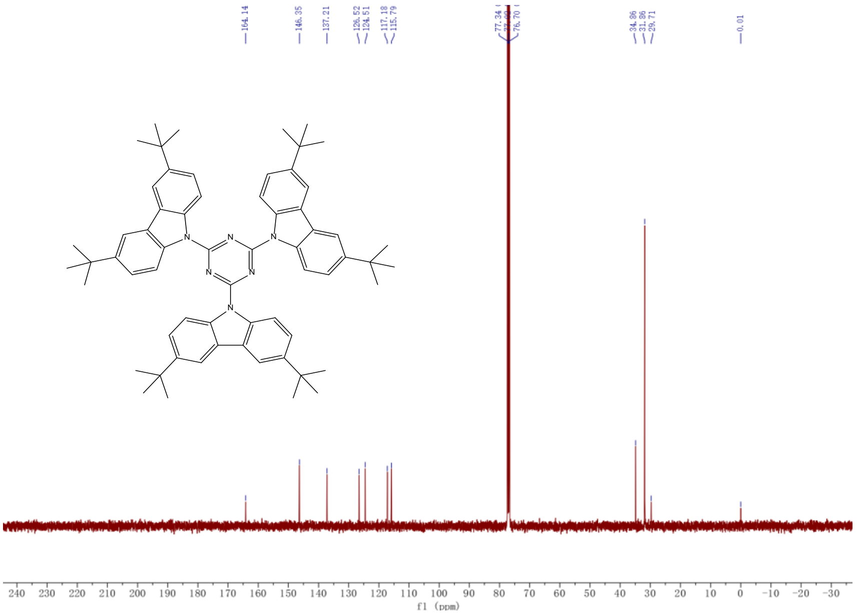

Figure S8. ^{13}CNMR spectrum of **TDBCZT** in $CDCl_3$.

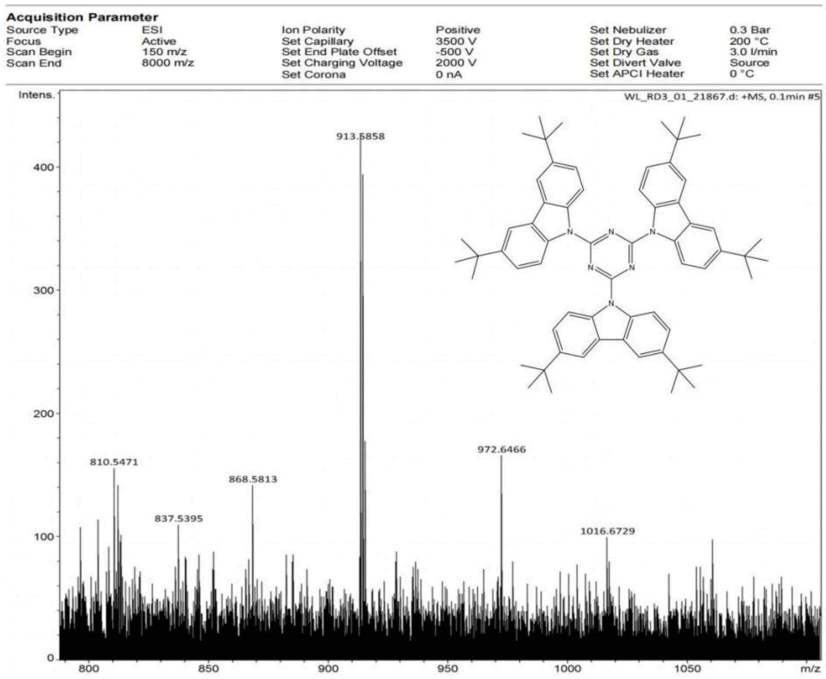

Figure S9. HRMS spectrum of **TDBCZT** in $CDCl_3$.

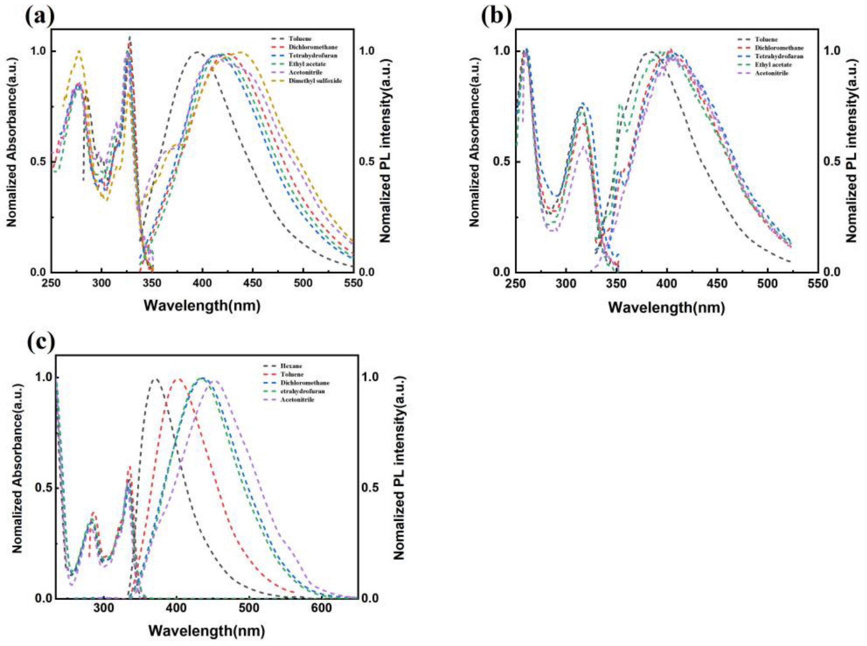

Figure S10. Normalized absorption and PL spectra of (**a**) **TCZT**, (**b**) **TIDT** and (**c**) **TDBCZT** in various solvents.

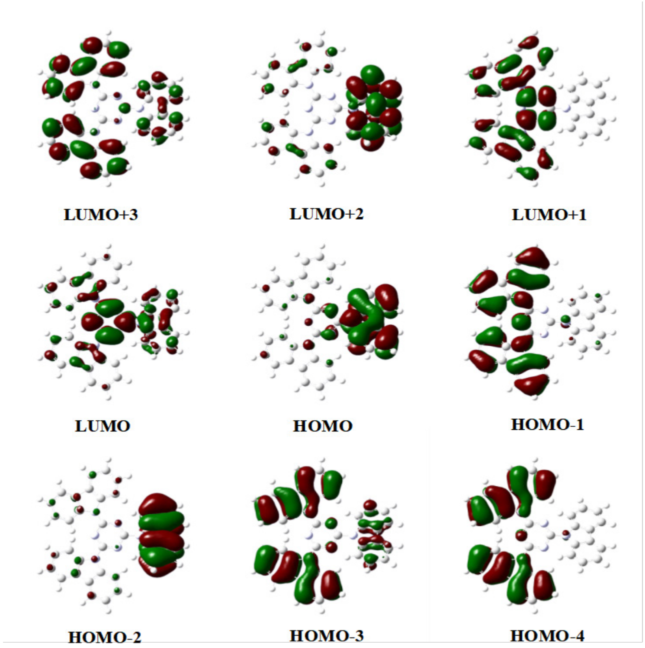

Figure S11. Frontier molecular orbitals associated with the electronic transitions of **TCZT**.

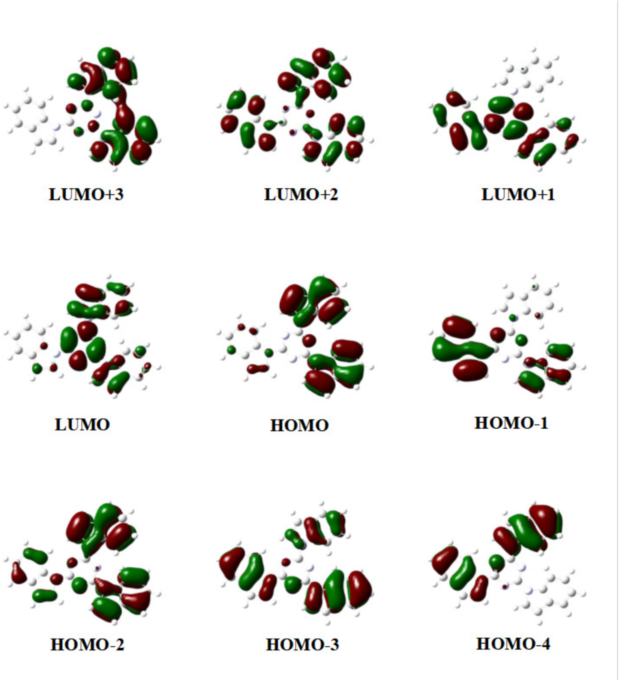

Figure S12. Frontier molecular orbitals associated with the electronic transitions of **TIDT**.

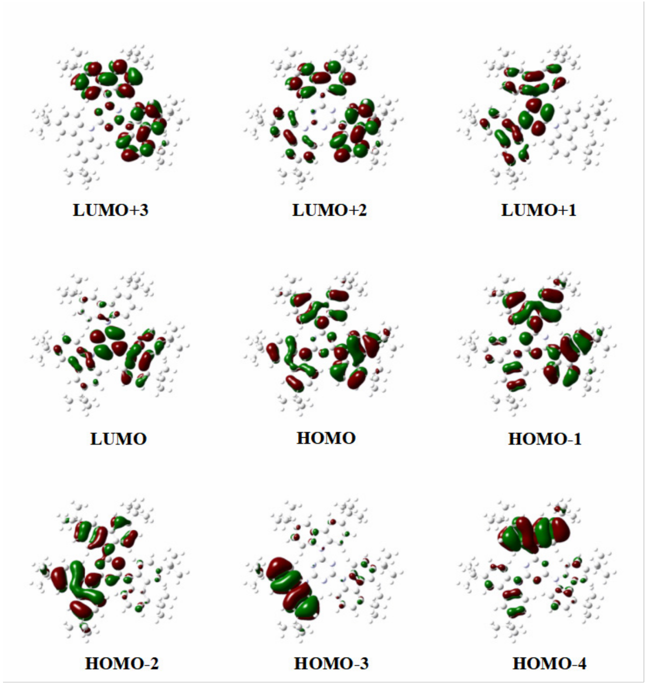

Figure S13. Frontier molecular orbitals associated with the electronic transitions of **TDBCZT**.

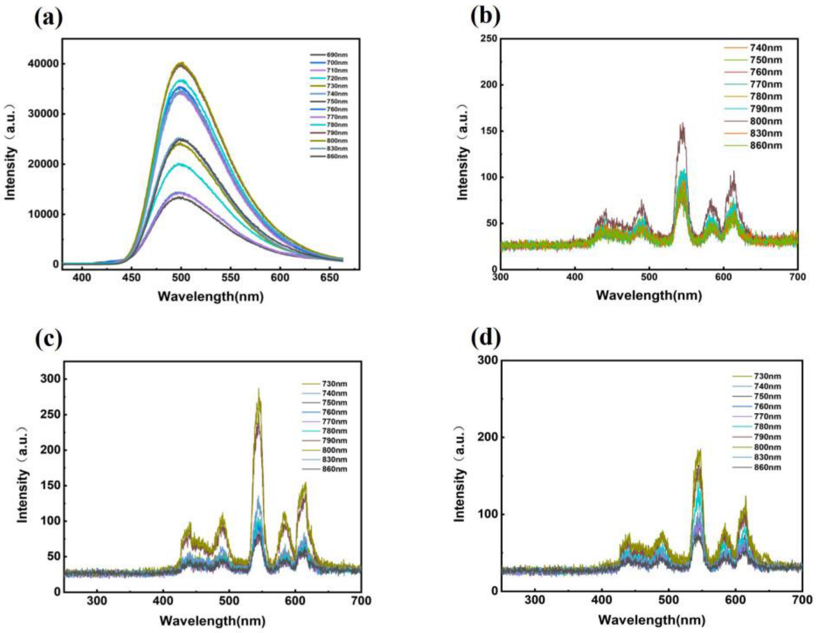

Figure S14. The TPEF spectra of (**a**) coumarin 307 (**b**) **TCZT** (**c**) **TIDT** (**d**) **TDBCZT** at various excitation wavelengths.

Table S1. The photophysical data of **TCZT** in various solvents.

Solvent	λ^{a}_{max} a) [nm]	ε b) [$10^4 M^{-1}cm^{-1}$]	λ^{e}_{max} c) [nm]	CIE(x,y) d)	Φ_f e) [%]
toluene	328	6.6	397	(0.155, 0.072)	46.0
DCM	328	6.1	422	(0.155, 0.108)	23.4
THF	327	6.7	412	(0.155, 0.094)	32.8
Ethyl acetate	326	7.0	417	(0.154, 0.099)	20.6
Acetonitrile	327	3.5	405	(0.157, 0.130)	25.0
DMSO	327	6.3	437	(0.156, 0.134)	27.2
Film	335	6.0	401	（0.171,0.112）	1.43

a) Absorption peaks in solutions (10^{-6} M) b) Molar absorptivity at maximum absorption wavelengths in solutions (10^{-6} M) c) Emission peaks in solutions (10^{-6} M) d) CIE coordinates e) Fluorescence quantum yields of **TCZT** at the maximum absorption wavelengths in solutions (10^{-6} M) using quinine sulfate in 0.1M H_2SO_4 solution (Φ =0.55) as reference and absolute fluorescence quantum yields measured in neat film state.

Table S2. The photophysical data of **TIDT** in various solvents.

Solvent	λ^{a}_{max} a) [nm]	ε b) [$10^4 M^{-1}cm^{-1}$]	λ^{e}_{max} c) [nm]	CIE(x,y) d)	Φ_f e) [%]
Toluene	317	12.1	381	(0.155, 0.067)	18.00
DCM	319	7.9	404	(0.156, 0.091)	13.00
THF	316	9.9	408	(0.155, 0.097)	13.60
Ethyl acetate	316	8.3	397	(0.156, 0.097)	7.40
Acetonitrile	327	7.9	403	(0.160, 0.135)	20.0
Film	331	15.2	381	(0.188, 0.145)	2.67

a) Absorption peaks in solutions (10^{-6} M) b) Molar absorptivity at maximum absorption wavelengths in solutions (10^{-6} M) c) Emission peaks in solutions (10^{-6} M) d) CIE coordinates e) Fluorescence quantum yields of **TIDT** at the maximum absorption wavelengths in solutions (10^{-6} M) using quinine sulfate in 0.1M H_2SO_4 solution (Φ =0.55) as reference and absolute fluorescence quantum yields measured in neat film state.

Table S3. The photophysical data of **TDBCZT** in various solvents.

Solvent	λ^{a}_{max} a) [nm]	ε b) [$10^4 M^{-1}cm^{-1}$]	λ^{e}_{max} c) [nm]	CIE(x,y) d)	Φ_f e) [%]
Hexane	336	72.8	371	(0.159, 0.061)	47.4
Toluene	336	59.8	402	(0.173, 0.995)	31.4
DCM	334	64.0	441	(0.157, 0.139)	22.4
THF	332	68.6	435	(0.155, 0.129)	23.2
Acetonitrile	332	77.2	450	(0.165, 0.185)	20.8
Film	331	15.2	370	(0.182, 0.146)	3.16

a) Absorption peaks in solutions (10^{-6} M) b) Molar absorptivity at maximum absorption wavelengths in solutions (10^{-6} M) c) Emission peaks in solutions (10^{-6} M) d) CIE coordinates e) Fluorescence quantum yields of **TDBCZT** at the maximum absorption wavelengths in solutions (10^{-6} M) using quinine sulfate in 0.1M H_2SO_4 solution (Φ =0.55) as reference and absolute fluorescence quantum yields measured in neat film state.

Table S4. Absorption wavelength and oscillator strength of **TCZT** evaluated by TD-DFT at the B3LYP/6-31G(d)

State	E_{gf} (eV)	λ_{gf} (nm)	f[a]	Transition[b]	Major contributions (%)
S_1	3.6929	335.74	0.4412	H→L	92
S_2	3.7914	327.01	0.0313	H→L+1	82
				H-3→L	3
S_3	3.8308	323.65	0.5109	H-1→L+1	90
				H→L+1	4
S_4	3.9108	317.03	0.0102	H-2→L	92
				H→L+1	2
S_5	3.9307	315.43	0.0004	H-1→L	72
				H-4→L	3
				H-2→L+1	3
S_6	3.9412	314.58	0.1716	H→L+1	10
				H-5→L	44
S_7	3.9656	312.65	0.027	H-4→L+1	61
S_8	3.9835	311.25	0.0481	H-4→L	44
S_9	4.0516	306.01	0.0001	H-2→L+1	39
S_{10}	4.0612	305.29	0	H-2→L+1	54
				H-3→L+1	34
S_{11}	4.0637	305.1	0.0083	H-3→L	39
				H-4→L+1	32
S_{12}	4.2073	294.69	0.0946	H-5→L+1	61
				H-4→L	9
T_1	3.1042	399.41	0	H-4→L+1	19
T_2	3.106	399.18	0	H-4→L+3	20
T_3	3.1586	392.52	0	H-2→L+2	52
T_4	3.3014	375.55	0	H→L	41
T_5	3.4062	363.99	0	H→L	39

[a] Oscillator strength. [b] H: HOMO, L: LUMO.

Table S5. Absorption wavelength and oscillator strength of **TIDT** evaluated by TD-DFT at the B3LYP/6-31G(d)

State	E_{gf} (eV)	λ_{gf} (nm)	f [a]	Transition [b]	Major contributions (%)
S_1	3.7214	333.16	0.2	H→L	90
S_2	3.8059	325.77	0.2782	H-1→L	77
S_3	3.8622	321.02	0.3239	H→L+1	34
				H-1→L+1	15
				H-1→L	11
S_4	3.8932	318.47	0.0099	H-2→L	48
				H→L+2	41
S_5	3.9343	315.14	0.0703	H-2→L+1	77
				H-1→L+4	8
S_6	3.9438	314.38	0.012	H-1→L+1	56
S_7	4.1895	295.94	0.116	H-3→L	61
				H-4→L	20
S_8	4.2088	294.58	0.2373	H-4→L	61
S_9	4.2535	291.49	0.1747	H-3→L+1	41
				H-4→L+1	5
S_{10}	4.2746	290.05	0.01	H-5→L	48
				H-4→L	3
S_{11}	4.3688	283.79	0.0686	H-4→L+1	82
				H-4→L	11
S_{12}	4.5026	275.36	0.0183	H-5→L+1	85
T_1	3.0171	410.94	0	H→L	42
T_2	3.0272	409.56	0	H-2→L	23
				H-1→L+1	30
T_3	3.04	407.84	0	H-1→L+1	14
T_4	3.4772	356.56	0	H-3→L	25
T_5	3.5853	345.82	0	H→L	22

[a] Oscillator strength. [b] H: HOMO, L: LUMO.

Table S6. Absorption wavelength and oscillator strength of **TDBCZT** evaluated by TD-DFT at the B3LYP/6-31G(d)

State	E_{gf} (eV)	λ_{gf} (nm)	f [a]	Transition [b]	Major contributions (%)
S_1	3.6623	338.54	0.5555	H→L	90
				H-2→L+1	4
				H-1→L	3
S_2	3.7209	333.21	0.5708	H→L+1	77
				H-2→L	10
S_3	3.7869	327.4	0.0234	H-1→L+1	41
				H-1→L	22
S_4	3.8545	321.66	0.129	H-2→L	16
				H-1→L+1	29
S_5	3.8721	320.2	0.0525	H-2→L	30
				H-1→L	27
S_6	3.9287	315.59	0.0497	H-3→L	48
				H-3→L+1	42
S_7	3.9401	314.67	0.0672	H-5→L	52
S_8	3.9703	312.28	0.0678	H-5→L+1	48
S_9	4.0112	309.1	0.0012	H-3→L+1	37
				H-2→L+1	6
S_{10}	4.0436	306.62	0.0001	H-4→L	58
T_1	3.0851	401.88	0	H-3→L+1	19
				H-1→L+4	32
T_2	3.102	399.69	0	H-5→L+2	22
T_3	3.1057	399.21	0	H-4→L+1	15
				H-4→L+2	16
T_4	3.2883	377.05	0	H-1→L	29
T_5	3.3442	370.74	0	H→L	50

[a] Oscillator strength. [b] H: HOMO, L: LUMO.
